# The successful posterior sectionectomy accompanied with caudate lobectomy for hepatocellular carcinoma located in segment 1 after LEN-TACE: a case report

**DOI:** 10.1007/s12328-024-01929-8

**Published:** 2024-02-14

**Authors:** Atsushi Nanashima, Takeomi Hamada, Masahide Hiyoshi, Naoya Imamura, Yuki Tsuchimochi, Ikko Shimizu, Kenji Nagata, Hiroshi Kawakami

**Affiliations:** 1 Division of Hepato-Biliary-Pancreas Surgery, Department of Surgery, Faculty of Medicine, University of Miyazaki, 5200 Kihara, Kiyotake, Miyazaki 889-1692 Japan; 2 Division of Gastroenterology and Hepatology, Department of Internal Medicine, Faculty of Medicine, University of Miyazaki, 5200 Kihara, Kiyotake, Miyazaki 889-1692 Japan

**Keywords:** Large hepatocellular carcinoma, Caudate lobe, Radical hepatectomy, Lenvatinib-transarterial chemoembolization sequential therapy, Conversion surgery

## Abstract

Nowadays, the novel molecular targeting chemotherapy provides possibility of safe hepatectomy for progressive hepatocellular carcinoma (HCC). Further, combination of the conventional transarterial chemoembolization (TACE) may add an effect of tumor shrink. We present a successful radical hepatectomy for a large HCC located in segment 1 accompanied with the preoperative Lenvatinib (LEN)-TACE sequential treatment. We present a woman patient without any complaints who had a 7 cm-in-size of solitary HCC compressing vena cava and right portal pedicle. To achieve radical hepatectomy by tumor shrinking, LEN-TACE for 2 months. After confirming downsizing or devascularization of the HCC, we scheduled radical posterior sectionectomy combined with caudate lobectomy according to tumor location and expected future remnant liver volume from three-dimensional computed tomography simulation before surgery. Under the thoraco-abdominal incision laparotomy, we safely achieved scheduled radical hepatectomy without any vascular injuries. The postoperative course was uneventful and no tumor recurrence were observed for 1 year. Histological findings showed the Japan TNM stage III HCC with 70% necrosis. The multi-modal strategy of LEN-TACE followed by radical hepatectomy by confirming downsizing or devascularization in tumor is supposed to be useful and would be a preoperative chemotherapy option, and promising for curative treatment in HCC patients with progressive or large HCC, which may lead to safety by prevention surrounding major vascular injury.

**Supplementary Information:**

The online version contains supplementary material available at 10.1007/s12328-024-01929-8.

## Introduction

Hepatocellular carcinoma (HCC) sometimes occurred in the segment 1 (caudate lobe of the liver) and the radical hepatectomy can be achieved in cases with small size without compression of surrounding vessels [1, 2]. In case of a large and rapidly progressive HCC, major vascular injuries as vena cava, hepatic veins or portal pedicles are highly expected which may lead to lethal situation during and after operation [3, 4]. Nowadays, the molecular targeting or immune-chemotherapy drugs such as Lenvatinib (LEN), or Atezolizumab plus Bevacizumab has been immediately developed in the field of treatments for unresectable or intermediate stage HCC, which might respond rapid tumor necrosis or shrinkage [5]. Furthermore, the reports of conversion surgery have been gradually increased at present [6]. Downsizing of HCC is required to secure important vessels or organ injury. LEN-transarterial chemoembolization (TACE) sequential therapy is also a good combination regimen by the combined cytocidal effects and devascularization for a large HCC [7].

We herein report a case of a large HCC located in segment 1 (caudate lobe) compressing surrounding vena cava and right lateral portal pedicle. To secure operative safety and preventing tumor rupture, LEN-TACE for 2 months followed by the conversion surgery of posterior sectionectomy with caudate lobectomy was achieved without tumor relapse for a year.

## Case report

**Fig. 1:**
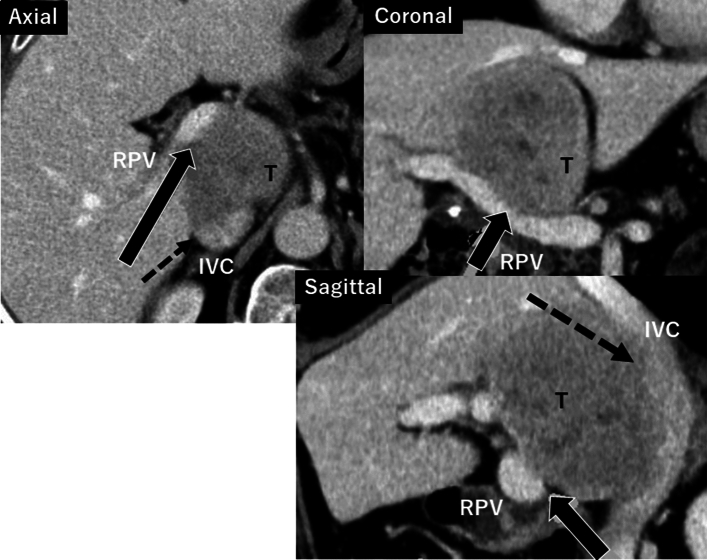
The solitary 6 cm-in-size of HCC was observed in the segment 1 by the enhanced CT findings. At the venous phase, the entire tumor enhancement was washed-out and compressed the portal trunk (black arrow), posterior portal pedicle and inferior vena cava (black dotted arrow), which was supposed to be vascular involvement

A case was the 82-year-old female and a 6 cm-in-size of liver cancer located in the segment 1 (caudate lobe) was found by the ultrasonography but no clinical complaints during the follow-up of hypertension, diabetes and chronic renal dysfunction. She underwent laparoscopic cholecystectomy for gallstone 12 years ago. She referred to the liver physicians and the tumor diagnosed as a solitary HCC (Fig. 1). At the venous phase of the enhanced CT showed an intra-tumorous washout finding which severely compressed the hepatic vena cava and the right portal pedicle. It seemed difficult to safely achieve radical surgery without unexpected massive bleeding due to major venous injury.

## Chemotherapy and TACE

**Fig. 2:**
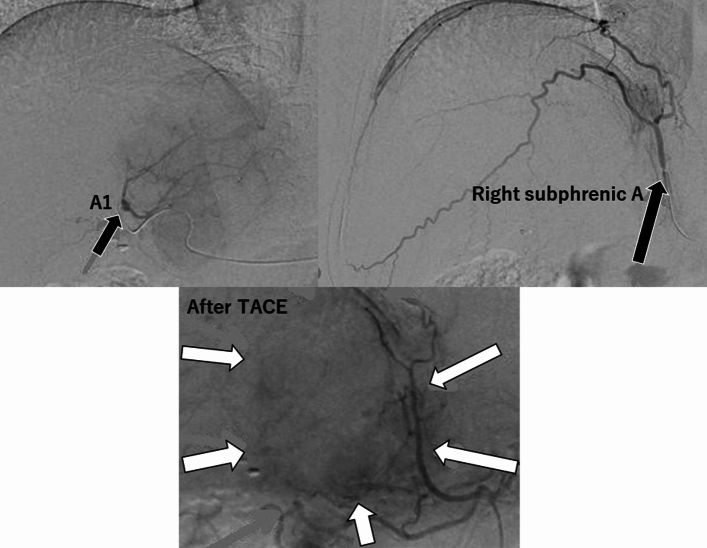
The finding of drug-eluting beads-TACE after 2cycles administration of Lenvatinib. The feeding arteries were segment 1 and right subphrenic arteries A from the celiac trunk. Just after the TACE, tumor vascularity was almost disappeared (white arrow area)

At the onset, the up-front surgery seemed to be risky of surrounding venous vascular injury and Lenvatinib chemotherapy was firstly induced. This strategy was discussed with liver physiologist and radiologist at the tumor board and the patient informed consent was given very carefully regarding side-effect of preoperative intervention and operative risk. Although adverse effects from LEN-TACE remained a concern, we still expected to achieve complete resection even with unexpected tumor growth or recovery from deterioration by the drug-induced temporally liver functional damage after cessation of the drug by tumor location. When good hepatic functional reserve improved for about 4 weeks in this patient, the patient provided informed consent for the LEN-TACE schedule and subsequent operation. Two cycles of oral LEN at 8 mg/day were administered in this case. At 2 months, however, the tumor size was still enlarged to 7 cm as a progressive disease state nevertheless of intra-tumorous vascularity was decreased. Therefore, the drug-eluting beads-TACE via segment 1 and the right subphrenic arteries was attempted (Fig. 2) twice for 2 months and, subsequently, 8 mg/day of LEN treatment was maintained for five cycles. Effects of downsizing (tumor shrink) to 5.5 cm was obtained and the hypo-vascularity of the tumor was confirmed. Eventually, the patient was again referred to our department of surgery and we decided the possibility of curability by the conversion surgery.

## Effect

**Fig. 3:**
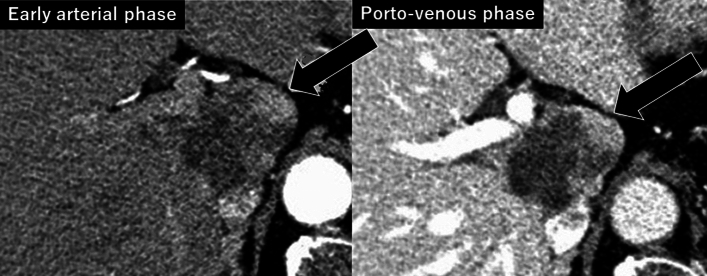
After LEN-TACE treatments for 7 months, HCC in the segment 1 was shrink as 5.5 cm and tumor compression was released but only posterior portal pedicle was involved as a stenosis by the follow-up CT. The tumor vascularity was almost disappeared at the arterial phase (black arrow)

A 5.8 cm-in-size of HCC was remained at the segment 1, which still compressed portal trunk, the right posterior portal vein, vena cava and the middle hepatic vein at the venous phase of the enhanced computed tomography (Fig. 3). Child–Pugh classification was A, the indocyanine green retention (ICG) rate at 15 min was 9.4%, and the uptake ratio between 3 and 15 min of ^99 m^-technethium galactosyl serum albumin liver scintigraphy was 0.959, which showed the good liver functional reserve before operation. The whole liver volume was 1137 cm^3^. The remnant liver volume was 63% (713 cm^3^) in case of left hepatectomy with caudate lobectomy, and that was 72% (811 cm^3^) in case of posterior sectionectomy with caudate lobectomy. ICGKrem value (ICG disappearance rate x remnant liver volume [8]) was calculated as 0.009 and 0.113, respectively. General function well preserved and the diabetes well controlled by the preoperative diet and 28 units of insulin administration daily.

## Surgical procedure

We decided the posterior sectionectomy with caudate lobectomy, because HCC might involve the right posterior portal pedicle and the better results of ICGKrem value.

**Fig. 4:**
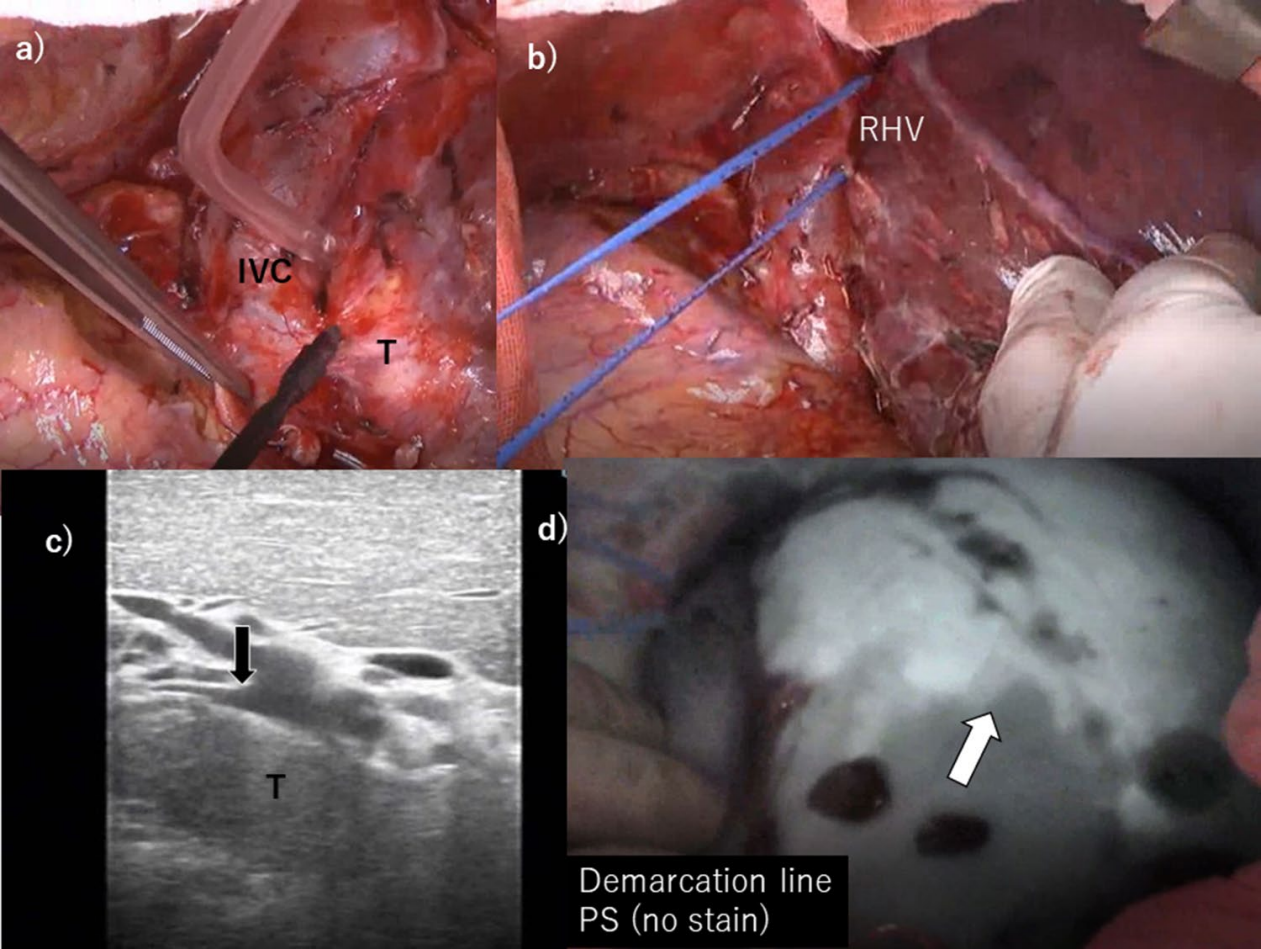
The intraoperative findings. **a** After mobilization of the right liver and cutting short hepatic veins, HCC in the segment 1 was gradually confirmed and adjacent to inferior vena cava (IVC) but not infiltration. **b**. The right hepatic vein (RHV) was secured and taped. **c** The intraoperative sonography showed the tumor (T) stenosis of the posterior portal pedicle and, however, it was possible to encircle the root of pedicle (black arrow). **d** After clamping the posterior portal pedicle, demarcation line of the posterior sector (PS) detected by the ICG photodynamic eye imaging (white arrow) was confirmed. Hepatic transection was started from this line

**Fig. 5:**
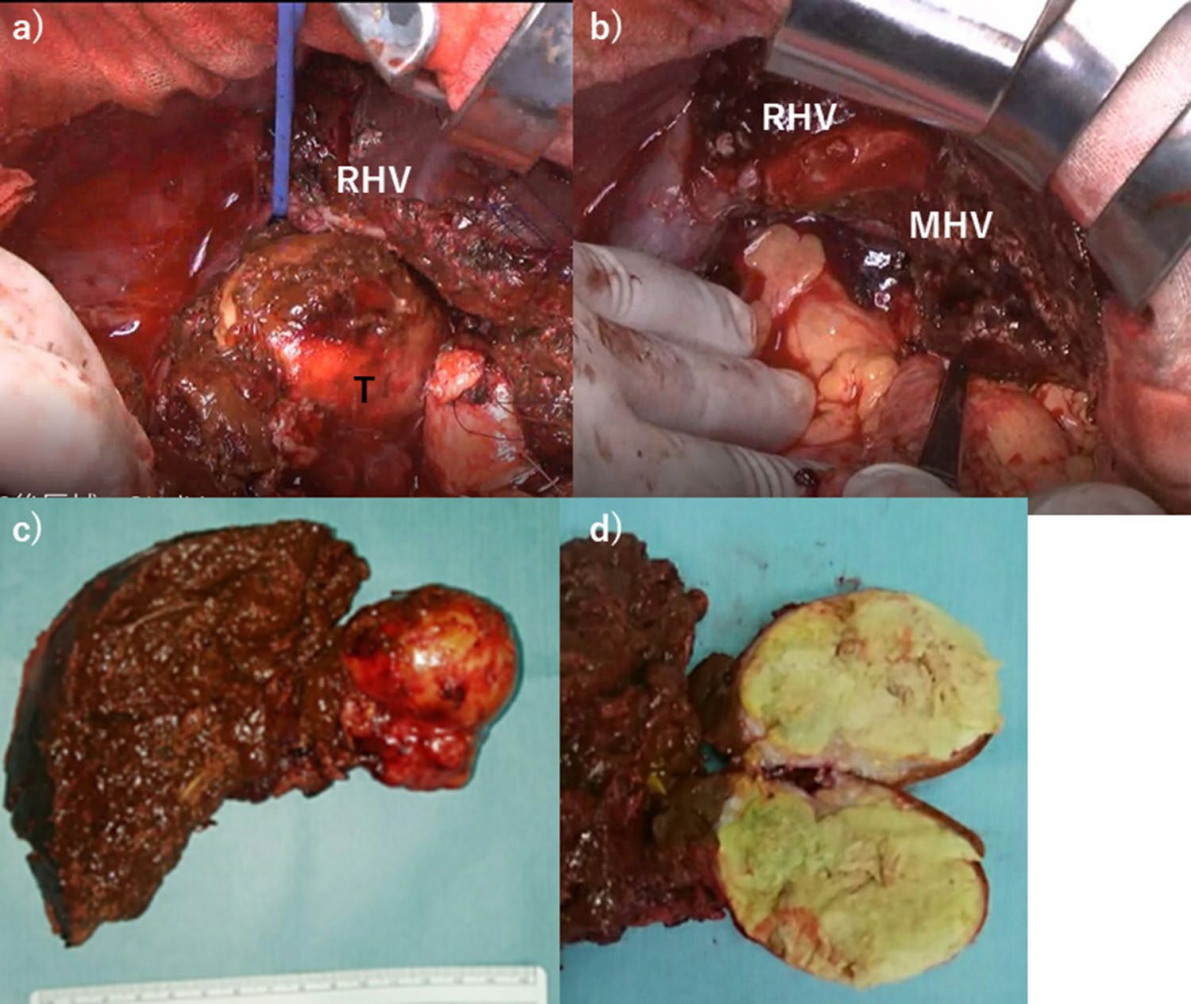
**a** Underneath RHV trunk, surrounding hepatic parenchyma of the caudate HCC was carefully transected by the clamp-crush procedure, **b** PS combined with caudate lobectomy was safely achieved and the compressed middle hepatic veins (MHV) was well maintained. **c** Resected specimen showed the capsulized HCC. **d** The intra-capsule tumor showed wide necrotic components

Under the thoraco-abdominal incisional approach (the seventh intercostal space) in the left lateral decubitus position, the right side liver was exposed. After fully mobilization of the right liver, the HCC in segment 1 was exposed after cutting short hepatic veins step-by-step (Fig. 4a). The intraoperative ultrasonography showed the tumor involvement of the right posterior portal pedicle, and severe compression to the middle hepatic vein but not infiltrated. Thus, the scheduled posterior sectionectomy combined with caudate lobectomy was maintained. After right hepatic vein was encircled, all short hepatic veins were transected between vena cava and caudate lobe although the connective tissue at the front of cava was edematous due to chemotherapy (Fig. 4b). Mobilization of the entire caudate lobe could be easily achieved due to tension-free palpation of devascularized HCC in the caudate lobe by the surgeon’s subjective touch feeling. The root of posterior portal pedicle was confirmed by the intraoperative sonography (Fig. 4c), which could be encircled and clamped by the extrahepatic approach. The negative staining area (demarcation line) of posterior sector were detected by the ICG photodynamic eye imaging (Fig. 4d). After transection of posterior sector, the combined caudate lobectomy containing HCC was carefully undergone underneath the right and middle hepatic veins (Fig. 5a, b). The remnant hepatic flows were well maintained without remarkable vascular injuries. The operating time was 358 min (the total inflow occlusion was 35 min), and blood loss was 440 mL but no red cell transfusion. (See supplementary video).

## Histology and postoperative course

Macroscopically, the resected specimen was 5.6 cm in size and necrotic tissue was remarkable (60–70% of coagulative necrosis; treatment effect grade 3 by the Rules of the Liver Cancer Study Group of Japan [9]) within the capsule (Fig. 5c, d). Microscopically, HCC was solitary, trabecular and solid type, no intrahepatic micro-metastasis, simple nodular type, expansive growth, capsular infiltration positive, s0, vp1, vv0, a0, b0 and pT3 [9]. Surgical margin negative (0.7 mm) and no hepatic fibrosis nor necroinflammatory findings. The postoperative course was uneventful, and she maintained tumor-relapse-free survival period for 12 months without adjuvant chemotherapy.

## Discussion

Treatments for the intermediate stage B by Barcelona Clinic Liver Cancer classification as our present case was established by chemotherapy or TACE in the western series [10]. However, our strategy has been decided the surgical removal if possible under the well tolerated functional liver reserve even though stage B HCC as well as other groups in Japan [11]. However, postoperative cancer-relapse-free or overall survivals in the advanced stage HCC have not been improved in the cases of hepatectomy alone. The useful adjuvant chemotherapy or radiological therapy has not been fully established for a long period. In the recent developing era of novel chemotherapy for HCC, paradigm-shift of multi-modal treatments is supposed to be important [12]. As in the present case of a large size HCC located at the caudate lobe, operative risks of massive bleeding surrounding major vascular or vena cava injuries by the upfront surgery are considered as our previous experience (not published in English), which are often lethal outcomes as postoperative hepatic failure. Morphological classification pattern involving portal pedicle and hepatic veins by a large HCC were classified by the Qiu’s classification system and the present case was closed to type IV characterized by tumor close proximity to or direct invasion of both portal and hepatic venous trunks [13]. In this report, type IV showed the highest risk of increased blood loss and the lethal postoperative complications. Furthermore, the type IV showed the shortest patient survivals due to above poor surgical results. Our previous study also showed that the increased blood loss by the major venous injuries has been related to the poor patient survivals [14]. Therefore, avoiding such an operative risk was supposed to be an associated surgical factor relating to curability. In such a case, it is speculated that the preoperative novel intervention as LEN-TACE may reduce surgical outcomes and lead better patient survival by tumor shrinkage or devascularization of HCC followed by hepatectomy. Moreover, spontaneous rapture is also concerned during waiting for operation. Therefore, we surgeons nowadays expected the prior control of tumor progression or tumor shrink to improve surgical results. Lenvatinib is one of promising drug for immediate HCC necrosis and this was firstly used for cancer control in this case [5–7]. However, the tumor size was enlarged after Lenvatinib treatment alone although the intra-tumorous necrosis was partially observed. Prior or sequential TACE combined with Lenvatinib (LEN-TACE) is a useful regimen by the stronger anti-HCC devascularization effect although clinical evidences of this schedule of prior LEN-TACE followed by the radical hepatectomy has not been fully elucidated by the clinical trial at this stage. Adequate mechanism by addictive anti-tumor effects of LEN-TACE regimen cannot be well explain in the present case, as a tumor slightly enlarged to 7 cm during Lenvatinib administration in the first step. According to the knowable information regarding these combination treatment, we speculate that the immunological and devascularization might be complemented each other to the HCC located in the caudate lobe, which is a specific location where uniform treatment effects of drug or embolization are difficult to obtain. However, fortunately, we achieved the good result of tumor shrinkage, wide intra-tumorous necrosis, good surgical record and over 1 year cancer-free period after hepatectomy even though portal and hepatic vein trunk were compressed. However, successful case reports have frequently been reported in virtual web conferences from several high-volume institutes (not published). In this format, liver surgeons have been able to discuss the advantages and adverse effects of LEN-TACE. We expected high therapeutic efficacy to be more likely than an unresectable or borderline situation based on the novel concept of Shindoh et al. for 8.4% of surgical intervention with good prognosis [6]. Thus, the sequential TACE twice showed quite a dramatic effect of tumor shrink in the present case and the operative indication was discussed after careful follow-up for tumor relapse, appearance of new regions or general status in this elderly patient. Atezolizumab plus bevacizumab combination therapy is also an alternative option and, however, it seems difficult to decide which is the first line for the neoadjuvant or conversion regimen at this stage [15]. If the operative possibility remains, our institute choice LEN-TACE first at present. After use of these regimens, the transient impairment of liver function is really concerned. It seems to show the immediate recovery to operable liver functional reserve after drug withdrawal. By the prior LEN-TACE, the mild inflammatory findings between HCC and vena cava was observed and the liver surface seemed to be fragile during operation. While, parenchymal transection, bleeding control could be safely performed, and the curative operation could be safely achieved. The decrease in tumor size was slight, but accompanied by intra-tumoral devascularization. Compression deformation had clearly changed within a short period after starting LEN-TACE. The risk of caval injury was thus considered to have been reduced. Although compression caused by HCC can usually be relieved in cases of simple nodular (encapsulated) lesions, we have previously encountered a case with laceration of the caval wall during exfoliation of a caudal HCC with inflammation or expansion by many collateral vessels [16]. Given that experience, we considered that some preparation in terms of tumor shrinkage is preferable for treating HCCs around major vessels. In fact, inflammation after TACE was not significant in the present case.

With respect to type of hepatectomy as posterior sectionectomy with caudate lobectomy, operative selection would be unusual but adequate option for such a present case. First reason is that the remnant liver volume was larger than left side hepatectomy and the posterior portal pedicle was consistently compressively involved by the HCC. Histological finding showed no tumor involvement at the main portal pedicle but tumor infiltration to the peripheral portal branch around the main tumor was observed. The second reason is that operative view around the vena cava and hepatic veins from the right dorsal side were good by the preoperative computerized simulation using the latest workstation software. This hepatectomy option for HCC located paracaval (right side) portion of caudate lobe were reported by a few congress abstracts in Japanese but not published yet. Imamura et al. reported the similar hepatectomy for atrophic posterior section with intrahepatic cholangiocarcinoma located at the paracaval portion in Japanese [17]. In the present case, HCC mainly located the central caudate lobe including Spiegel lobe. This operative view seemed to be better in comparison with the anterior transection approach. Further, the Takayama’s highly dorsal caudate lobectomy would be difficult for such a large HCC [2]. Thus, we propose the present selection of hepatectomy for caudate HCC as a useful option. By the tumor shrink, it would be easier to dissect from the surrounding major vessels. Macroscopic and histological findings showed the high rate of tumor necrosis within the tumor capsule. LEN-TACE might provide satisfied operative feasibility and short-term patient prognosis in this case. The present incision for posterior sectionectomy was supposed to be an alternative option as described by Imamura et al. [17] and, under a right-side view from the thoracoabdominal approach, the entire vena cava was easily confirmed. Thus, the injured part seemed to be fixed with this view and we could approach Spiegel’s caudate lobe by placing spacers like large towels on the left side cavity.

In conclusion, the combined and sequential treatment of LEN-TACE is supposed to be one of preoperative useful options to control cancer progression to aim the neoadjuvant or conversion treatment strategy for a large size of HCC located in the segment 1 although our consideration of usefulness for local control by LEN-TACE option before operation would be overemphasized at this stage. The multi-modal strategy will be still developed in various situation in the locally advanced stage HCC by more accumulation of oncological evidences as a preoperative adjuvant option. In the era of such novel strategies for advanced HCC, decisions on treatment protocols should be made in collaboration among not only surgeons, but also physicians, radiologists or other liver experts similar to functioning cancer boards. By the surgical point of view, the combined posterior sectionectomy with caudate lobectomy is feasible and safe operative option for the caudate HCC under the precise computerized simulation before surgery.

### Supplementary Information

Below is the link to the electronic supplementary material.

Supplementary file1 (WMV 39179 KB)

## Abbreviations

HCC: Hepatocellular carcinoma
LEN-TACE: Lenvatinib-transarterial chemoembolization
ICG: Indocyanine green
